# A novel method for gathering and prioritizing disease candidate genes based on construction of a set of disease-related MeSH® terms

**DOI:** 10.1186/1471-2105-15-179

**Published:** 2014-06-10

**Authors:** Toshihide Ono, Satoru Kuhara

**Affiliations:** 1 Department of Genetic Resources Technology, Faculty of Agriculture, Kyushu University, 6-10-1 Hakozaki Higashi-ku, Fukuoka 812-8581, Japan; 2 Institute of Biomedical Innovation, Otsuka Pharmaceutical Co. Ltd., 463-10 Kagasuno Kawauchi-cho, Tokushima 771-0192, Japan

## Abstract

**Background:**

Understanding the molecular mechanisms involved in disease is critical for the development of more effective and individualized strategies for prevention and treatment. The amount of disease-related literature, including new genetic information on the molecular mechanisms of disease, is rapidly increasing. Extracting beneficial information from literature can be facilitated by computational methods such as the knowledge-discovery approach. Several methods for mining gene-disease relationships using computational methods have been developed, however, there has been a lack of research evaluating specific disease candidate genes.

**Results:**

We present a novel method for gathering and prioritizing specific disease candidate genes. Our approach involved the construction of a set of Medical Subject Headings (MeSH) terms for the effective retrieval of publications related to a disease candidate gene. Information regarding the relationships between genes and publications was obtained from the gene2pubmed database. The set of genes was prioritized using a “weighted literature score” based on the number of publications and weighted by the number of genes occurring in a publication. Using our method for the disease states of pain and Alzheimer’s disease, a total of 1101 pain candidate genes and 2810 Alzheimer’s disease candidate genes were gathered and prioritized. The precision was 0.30 and the recall was 0.89 in the case study of pain. The precision was 0.04 and the recall was 0.6 in the case study of Alzheimer’s disease. The precision-recall curve indicated that the performance of our method was superior to that of other publicly available tools.

**Conclusions:**

Our method, which involved the use of a set of MeSH terms related to disease candidate genes and a novel weighted literature score, improved the accuracy of gathering and prioritizing candidate genes by focusing on a specific disease.

## Background

Understanding the molecular mechanisms involved in disease is critical for the development of more effective and individualized strategies for prevention and treatment. At present, there are several strategies available for gathering appropriate information about the molecular mechanisms of disease from a genetic point of view. One of these strategies is to search specific disease-related gene databases [1,2]. As these databases are built based on manual processes, the data quality is high. However, owing to high manpower costs, it is difficult to maintain these databases with the large volume of ever-growing new literatures.

The other strategy is to use bioinformatics methods to extract and prioritize disease candidate genes automatically [3,4]. These methods have been classified into several categories, such as text mining and integration of multiple data sources. Text mining methods have been particularly well studied in the biological field, because scientific literature represents a rich source for mining-based retrieval of information on gene-disease relationships. Some methods for extracting knowledge from literature are based on keyword co-occurrence analysis and the automatic extraction of entity names from text [5]. The ranking method of gene prioritization is based on the co-occurrence of query terms, association words, and database terms, and a rule-based pattern recognition algorithm. Examples of these tools include PolySearch [6] and LEGENDA [7]. In addition, methods to extract and prioritize candidate genes based on multiple data sources, such as sequence-based features, annotation data and GWAS data, have been proposed. Examples of these tools include Genotator [8] and Gene Prospector [9]. While several attempts have been made to comprehensively extract and prioritize candidate disease genes by using bioinformatics techniques, a methodology for determining specific disease-relevant genes, such as pain, has not yet been fully developed [10].

The aim of our study was to develop a semi-automated method of gathering and prioritization of specific disease-related genes using a specialist’s knowledge that is GO and MP term selection. In this study, our approach has been applied to pain disease. In addition, we conducted a gene search related to Alzheimer’s disease (AD) to evaluate the application of this method to other specific disease. There are significant unmet medical needs in both diseases. To understand the underlying molecular mechanism is important to develop the new treatment and drug.

Our method involved the following: avoiding errors associated with gene name recognition using the gene2pubmed database [11], which links to PubMed® literature related to the gene, improving information retrieval from PubMed by creating a set of pain-related MeSH terms, and improving the prioritization accuracy with a novel score based on the number of publications. The priority score is weighted by the number of genes occurring in a publication. The performance of our method was compared to those of other publicly available tools. We will illustrate our method and present the results of its performance.

## Methods

### Data sources

Literature data available in July 2012 was obtained from the NLM (National Library of Medicine). MeSH terms were also obtained from NLM (http://www.nlm.nih.gov/mesh/meshhome.html). GO terms [12] were downloaded from the Gene Ontology Consortium web site (http://www.geneontology.org/). Mammalian phenotype (MP) terms [13] were downloaded from the Mouse Genome Informatics (MGI) website (http://www.informatics.jax.org/searches/MP_form.shtml). The list of pain-related genes was downloaded from the Pain Gene Database website (http://www.jbldesign.com/jmogil/enter.html) [14]. The list of AD-related genes was constructed from OMIM and KEGG database [15,16].

### Validation sets

Two sets of disease-related genes were generated to assess the performance of the method for each disease, those were pain and AD. The first set, gene set 1, was used to assess the performance of the disease-related MeSH terms and prioritizing score. The other set, gene set 2, was used to compare the performance of our method to a simple approach based on the number of the gene-related publications, resulting from the search keyword, for example, “pain” or “pain [MeSH]”, as well as with other publicly available tools.

Gene set 1 was constructed from GO and MP data. The relationship between the GO terms and genes were obtained from the AmiGO website [17]. The relationship between MP terms and genes were obtained from the MGI website. Pain- or AD-related GO and MP terms, which were manually selected by specialists in the fields of pain or AD, are summarized in Tables 1 and 2. Gene set 1 included 308 genes for pain (pain gene set 1), and 123 for AD (AD gene set 1). Gene set 2 for pain (pain gene set 2) was constructed from the Pain Gene Database. This set contained 369 unique, manually curated genes as of September 2012. Gene set 2 for AD (AD gene set 2) was constructed from OMIM (#104300) and KEGG (hsa05010) databases. This set contained 123 unique genes. These databases which were source of the gene set 2 were used as the evaluation set in other studies for gene prioritization [18-20].

**Table 1:** Lists of a) gene ontology terms and b) mammalian phenotype terms that were used to create pain gene set 1

**ID**	**Term**
a)	
GO:0019233	Sensory perception of pain
GO:0050968	Detection of chemical stimulus involved in sensory perception of pain
GO:0050967	Detection of electrical stimulus involved in sensory perception of pain
GO:0050966	Detection of mechanical stimulus involved in sensory perception of pain
GO:0050965	Detection of temperature stimulus involved in sensory perception of pain
GO:0051930	Regulation of sensory perception of pain
GO:0044465	Modulation of sensory perception of pain in another organism
GO:0019234	Sensory perception of fast pain
GO:0019235	Sensory perception of slow pain
GO:0048265	Response to pain
GO:0048266	Behavioral response to pain
GO:0061366	Behavioral response to chemical pain
GO:0061367	Behavioral response to acetic acid induced pain
GO:0061368	Behavioral response to formalin induced pain
b)	
MP:0001491	Unresponsive to tactile stimuli
MP:0001968	Abnormal touch/nociception
MP:0001970	Abnormal pain threshold
MP:0001973	Increased thermal nociceptive threshold
MP:0001980	Abnormal chemically-elicited antinociception
MP:0001981	Increased chemically-elicited antinociception
MP:0001982	Decreased chemically-elicited antinociception
MP:0002733	Abnormal thermal nociception
MP:0002734	Abnormal mechanical nociception
MP:0002735	Abnormal chemical nociception
MP:0002736	Abnormal nociception after inflammation
MP:0002738	Hyperresponsive to tactile stimuli
MP:0003043	Hypoalgesia
MP:0003177	Allodynia
MP:0003998	Decreased thermal nociceptive threshold
MP:0004270	Analgesia
MP:0005316	Abnormal response to tactile stimuli
MP:0005407	Hyperalgesia
MP:0005498	Hyporesponsive to tactile stimuli
MP:0008531	Increased chemical nociceptive threshold
MP:0008532	Decreased chemical nociceptive threshold

**Table 2:** Lists of a) gene ontology terms and b) mammalian phenotype terms that were used to create AD gene set 1

**ID**	**Term**
a)	
GO:0001540	Beta-amyloid binding
GO:0034205	Beta-amyloid formation
GO:0034231	Slet amyloid polypeptide processing
GO:0042982	Amyloid precursor protein metabolic process
GO:0042983	Amyloid precursor protein biosynthetic process
GO:0042984	Regulation of amyloid precursor protein biosynthetic process
GO:0042987	Amyloid precursor protein catabolic process
GO:0044548	S100 protein binding
GO:0048152	S100 beta biosynthetic process
GO:0048153	S100 alpha biosynthetic process
GO:0048156	Tau protein binding
GO:0050435	Beta-amyloid metabolic process
GO:0097242	Beta-amyloid clearance
GO:1900221	Regulation of beta-amyloid clearance
GO:1902003	Regulation of beta-amyloid formation
GO:1990000	Amyloid fibril formation
b)	
MP:0000604	Amyloidosis
MP:0008493	Alpha-synuclein inclusion body
MP:0003214	Neurofibrillary tangles
MP:0004250	Tau protein deposits

### Overview of the method for the gathering and prioritizing of disease candidate genes

**Figure 1:**
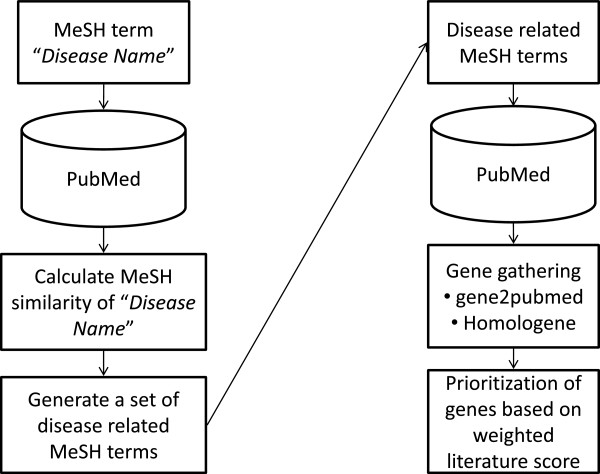
**Flowchart of the method for gathering and prioritizing pain candidate genes.** We initially created a set of MeSH terms for comprehensive retrieval of disease-related publications (left). Next, specific disease candidate genes were obtained from disease-related publications, which were searched for with a set of disease-related MeSH terms. Finally, the prioritizing score was calculated based on the weighted literature score (right).

The overall architecture of our method for the gathering and prioritizing of each gene is shown in Figure 1. We first optimized the search keywords for the comprehensive retrieval of the publications related to disease candidate gene. We calculated the occurrence similarity between the MeSH term “*disease name*”, such as “pain” or “Alzheimer’s disease”, and other MeSH terms to retrieve the MeSH terms related MeSH terms “*disease nam*e” which were defined as “disease-related MeSH terms”. Then we retrieved publications using disease-related MeSH terms from PubMed. The relationships between these publications and genes were obtained from the gene2pubmed database. Finally, a prioritizing score was calculated from the occurrence-based literature score to predict the gene most likely to be related to the disease. The following sections describe the methods in more detail.

### Obtaining of the relationship between genes and publications

The relationship between genes and publications was collected with gene2pubmed databases. The gene2pubmed database contains curated associations between the NCBI Gene and PubMed databases. Gene2pubmed database links to publications for each gene and is not limited to articles specifically addressing the function of gene. We retrieved gene information from publications resulting from a search for disease-related MeSH terms.

We investigated orthologous gene pairs using the Homologene database [21] to unify the redundancy of genes for multiple species. In this study, genes from human, mouse, and rat species were considered, because the majority of studies in the field of pain and AD research is conducted in these species.

### Selection of disease-related MeSH terms

To achieve comprehensive retrieval of publications related to a disease candidate gene, we constructed an optimal set of MeSH terms by calculating the frequency of co-occurrence between the MeSH terms for the disease states of “pain” or “Alzheimer disease”, and other MeSH terms.

The occurrence similarity between these terms in publications which were referred in the gene2pubmed database was calculated using cosine similarity, Dice similarity, Jaccard similarity, mutual information, and Simpson similarity measures.

The cosine similarity measure was computed as follows:

$$\mathrm{si}m_{\mathrm{cosine}}\left(X,Y\right)=\frac{\left|X\cap Y\right|}{\sqrt{\left|X\right|\left|Y\right|}}$$

The Dice similarity measure was computed as follows:

$$\mathrm{si}m_{\mathrm{Dice}}\left(X,Y\right)=\frac{2\left|X\cap Y\right|}{\left|X\right|+\left|Y\right|}$$

The Jaccard similarity measure was computed as follows:

$$\mathrm{si}m_{\mathrm{Jaccard}}\left(X,Y\right)=\frac{\left|X\cap Y\right|}{\left|X\cup Y\right|}$$

The Simpson similarity measure was computed as follows:

$$\mathrm{si}m_{\mathrm{Simpson}}\left(X,Y\right)=\frac{\left|X\cap Y\right|}{\min\left(\left|X\right|,\left|Y\right|\right)}$$

The point-wise mutual information measure was computed as follows:

$$\mathrm{PMI}\left(X,Y\right)=\frac{\log P\left(X,Y\right)}{P\left(X\right)P\left(Y\right)}$$

Where |*X*| refers to the number of publications with the MeSH term for the disease and |*Y*| refers to the number of publications with another MeSH term. |*X* ∩ *Y*| refers to the number of publications with the MeSH term for the disease co-occurring with another MeSH term. *P(X,Y)* is the probability that *X* and *Y* elements appear at the same time. *P(X)* and *P(Y)* are the probabilities of occurrence of each element.

We created multiple sets of MeSH terms, starting with the most closely related terms and incrementally extending the list by one term. For example, using cosine similarity analysis in the case study of pain, the first set included MeSH terms “Pain” and “Pain Measurement”, and the second set included MeSH terms “Pain”, “Pain Measurement”, and “Nociceptors” (Table 3). We searched PubMed using each set of MeSH terms, which were combined with “OR” operators using the “[MeSH:NoExp]” option.

**Table 3:** Example of pain-related MeSH terms obtained on the basis of the cosine similarity

**Mesh term**	**MeSH category**	**Cosine distance**	**MeSH term set**									
				**#1**	**#2**	**#3**	**#4**	**#5**	**#6**	**#7**	**#8**	**#9**
Pain	C, F, G	1.000	x	x	x	x	x	x	x	x	x	x
Pain measurement	E	0.340		x	x	x	x	x	x	x	x	x
Nociceptors	A	0.268			x	x	x	x	x	x	x	x
Pain threshold	F, G	0.259				x	x	x	x	x	x	x
Hyperalgesia	C	0.225					x	x	x	x	x	x
Posterior horn cells	A	0.180						x	x	x	x	x
Ganglia, spinal	A	0.147							x	x	x	x
Injections, spinal	E	0.147								x	x	x
Physical stimulation	E	0.146									x	x
Formaldehyde	D	0.145										x
Recall			0.669	0.692	0.693	0.693	0.703	0.700	0.668	0.667	0.661	0.650

The relationships between genes and publications were obtained using the method previously described. The accuracy of the obtained gene list was evaluated using recall at rank which was the number of genes obtained in publications using the query “pain [MeSH:NoExp]” or “Alzheimer Disease [MeSH:NoExp]”. The set of MeSH terms that achieved the highest recall was defined as the set of “pain-related MeSH terms” or “AD-related MeSH terms”.

### Calculation of the prioritizing score

To prioritize the genes in the gene set, we introduced a “weighted literature score”, which was based on the number of the genes referred to by a publication. This score depends on the assumption that if a publication referred to many genes, the degree of contribution to each gene by that study is low. For example, publications describing microarray or genome sequencing studies refer to many genes so we assumed that the contribution of these publications to each gene was relatively minor. Therefore, we defined the weighted literature score of gene *i*, which was denoted as *WLSi*, using the following equation:

WLSi=∑j=1nfi,j×1Pj

Where *n* refers to the total number of publications associated with the gene2pubmed database, and *Pj* refers to the number of genes associated with publication *j. f*(*i*, *j*) = 0 if gene *i* is not associated with publication *j* and *f*(*i*, *j*) = 1 if gene *i* is associated with publication *j*.

To assess the performance of this scoring method, we compared it against three other well-known scoring methods based on the number of gene-related publications. The measures used for comparison were the number of publications, modified TF-IDF measure, and p-value calculated with the hypergeometric distribution and Benjamini-Hochberg false discovery rate correction [22].

The modified TF-IDF measure score of gene *i* was denoted as *TFIDFi* and determined using the following equation:

TFIDFi=∑j=1nfi,j×lognLi

*Lj* refers to the number of publication associated with gene *i*.

The recall of each rank of genes from each scoring method was calculated against gene set 1. The Wilcoxon signed-rank test was used to calculate the statistical significance of prioritization.

### Performance assessment of gene prioritization

To assess the performance of gene prioritization, we calculated the accumulated precision, recall, and F-measure for the top *n* out of the total genes in the ranking. In this approach, *Tn* represents the number of genes retrieved by our method or other publicly available tools that have been extracted correctly in the top *n* genes, which were included in gene set 2. *C* is the total number of genes in gene set 1 or gene set 2. *En* is the number of genes extracted by our method or other publicly available tools in the top *n* genes.

We defined recall, which was denoted as *R(n)*, precision, which was denoted as *P(n)*, and the F-measure (i.e., harmonic average of precision and recall), which was denoted as *F(n)* in the top *n* genes, based on the following equations:

$$R\left(n\right)=\frac{\mathrm{Tn}}{C}$$

$$P\left(n\right)=\frac{\mathrm{Tn}}{\mathrm{En}}$$

$$F\left(n\right)=\frac{2\;R\left(n\right)\;P\left(n\right)}{R\left(n\right)+P\left(n\right)}$$

The maximum F-measure was also used to compare to performance of gene prioritization.

### Comparing the performance against other keywords

We compared the performance of our method to a simple approach based on the ranking of the genes according to the number of gene related publications, resulting from the search term “pain” or “pain [MeSH]” in the case study of pain. First, we retrieved publications from PubMed using the keyword “pain” or “pain [MeSH]”. Next, the relationships between these publications and genes were obtained from the gene2pubmed database. Then, a prioritizing score was calculated from the number the gene related publications. Finally, for each method we compared the values of accumulated precision, recall, and maximum F-measure for the top *n* out of the total genes in the ranking. We also conducted this comparison for AD.

### Comparing the performance against other publicly available tools

We compared the performance of our method against several publicly available tools which extract the general gene-disease relationship by using various methods, which are text mining and integration of multiple data sources, in order to assess the effect of focusing on pain field. PolySearch and LEGENDA extract knowledge data from the literature based on keyword co-occurrence analysis and automatic extraction of entity names from text. Genotator and Gene Prospector extract and prioritize candidate genes based on multiple data sources.

The prioritization lists of pain or AD candidate genes identified using other tools were compiled using queries of the keyword “pain” or “Alzheimer’s disease”, and default parameters as of September 2012. In PolySearch, genes with the description “protein family or complex” were excluded because it was not possible to determine the relationships between genes and gene symbols within the NCBI Gene database. As these tools are limited to human genes, orthologous information was incorporated by the Homologene database. We compared the value of the accumulated precision, recall, and maximum F-measure for the top *n* out of the total genes in the ranking.

## Results

### Case study 1: pain

Pain is a major healthcare issue as defined by the World Health Organization and has complex pathophysiology [23,24]. Especially, chronic pain has remained a major healthcare issue affecting not only patients by decreasing their quality of life but also society as a whole by increasing socioeconomic costs. Unfortunately, many pathological pain conditions remain poorly understood and resist currently available treatment. Therefore, the development of new therapeutic approaches to managing pain will undoubtedly depend on a better understanding of the molecular, cellular, and circuit mechanisms underlying pain states.

In this study, genes related to primary disease, such as diabetes mellitus, cancer, and infectious disease were not considered.

### Evaluation of scoring methods for gene prioritization

**Figure 2:**
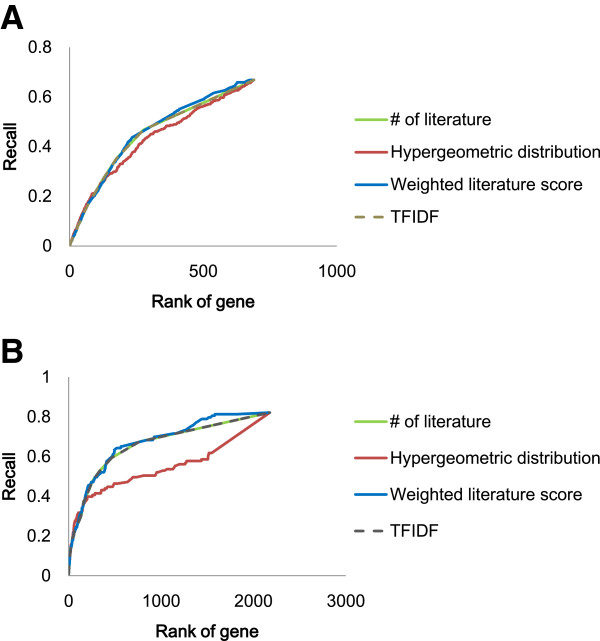
**Comparison of prioritization score performance. A)** Pain case study **B)** AD case study.

We initially evaluated the performance of scoring methods for gene prioritization because we used the best scoring method for constructing a set of pain-related MeSH terms. A total of 690 genes were obtained from the publications searched using the keyword MeSH term “pain [Mesh:NoExp]” in PubMed. The prioritization scores of these genes were calculated using four methods and the recall of each method is shown in Figure 2A. The weighted literature score produced the best performance (p < .05). The higher ranked genes were predicted accurately with each method. The weighted literature score achieved better prediction accuracy at the low ranking area than other methods.

Therefore, for subsequent analyses, the weighted literature score was applied for prioritization.

### Selection of pain-related MeSH terms

**Figure 3:**
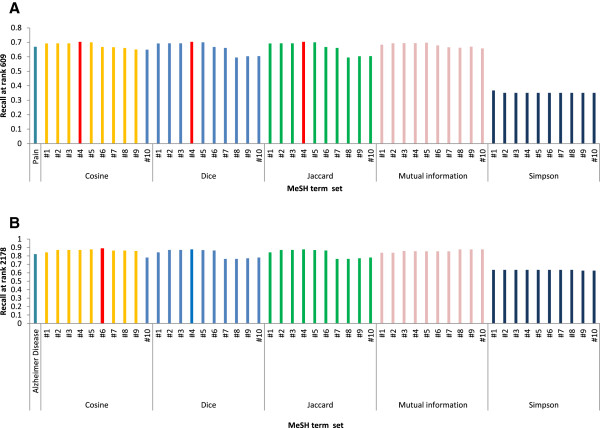
**Summary of the recall of prioritized genes resulting from each set of MeSH terms constructed using various similarity measures. A)** Recall was calculated against pain gene set 1. “Pain” is located in the leftmost means the recall of the list of genes constructed by searching for the keyword “pain [Mesh:NoExp]” **B) **The recall was calculated against AD gene set 1. “Alzheimer Disease” is located in the leftmost means the recall of the list of genes obtained by searching for the keyword “Alzheimer Disease [Mesh:NoExp]”. The red bars indicate the set of MeSH terms with the highest recall.

Figure 3 shows the recall of the genes obtained from publications retrieved from searches for each set of MeSH terms created by the various similarity measures. The highest recall (0.703) was achieved using MeSH term set 4 which was constructed using MeSH term similarity, which was calculated by the cosine, Dice, and Jaccard measures against pain gene set 1. This set comprised the combination of the MeSH terms “pain”, “pain measurement”, “nociceptor”, “pain threshold”, and “hyperalgesia”. These five MeSH terms were defined as pain-related MeSH terms.

Table 3 shows that the example of MeSH terms with a high occurrence similarity with the MeSH term “pain” calculated by the cosine similarity and the recall of gathering genes. MeSH terms with close cosine distance to the MeSH term “pain” belonged to various categories of MeSH tree structures (Table 3). Some of these categories are different from the categories that the MeSH term “pain” belongs to, which are “C” (Disease), “F” (Psychiatry and Psychology), and “G” (Phenomena and Processes). The MeSH term with the closest cosine distance value (0.34) was “Pain Measurement” which belongs to category “E” (Analytical, Diagnostic, and Therapeutic Techniques and Equipment). The term with the second closest cosine distance value (0.268) was “Nociceptors” which belongs to category “A” (Anatomy).

### Gathering and prioritizing the pain candidate genes

**Figure 4:**
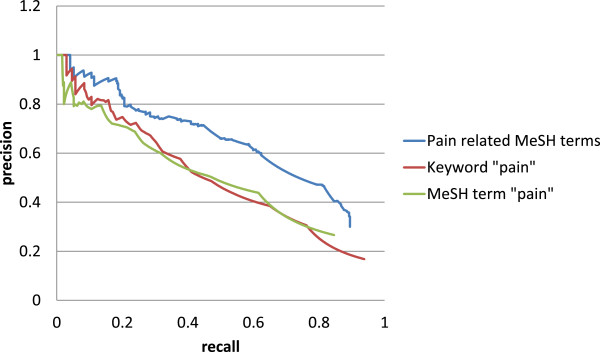
**Comparison of pain candidate gene prioritization performance.** Precision-recall plots show the performance of our method and of a simple approach based on the ranking of genes according to the number of gene related publications, resulting from the search term “pain” or “pain [MeSH]”.

To assess the performance of our method, precision and recall were compared at each gene rank for three gene sets against pain gene set 2. These sets were created using publications resulting from searches for pain-related MeSH terms, “pain” and “pain [MeSH]” in Pubmed.The precision-recall curves for each set are presented in Figure 4. Our method achieved a precision value of 0.30 and a recall value of 0.89 for 1101 genes. The precision and recall values for 2059 genes from simple approach based on the ranking of the genes by the number of times the gene appears with the keyword “pain” were 0.17 and 0.93, respectively, whereas the precision and recall values for 1172 genes from simple approach with the keyword “pain [MeSH]” were 0.27 and 0.84, respectively. The maximum F-measures for pain-related MeSH terms, “pain” and “pain [MeSH]” were 0.61, 0.48, and 0.51 with gene ranks of 381, 624, and 518, respectively. We found the performance of our method to be superior to alternative simple methods, with a higher precision-recall curve across the entire range.

The top 20 ranked genes from our method are summarized in Table 4 (all genes are summarized in Additional file 1). The top ranked gene was *TRPV1* (WLS = 140.92), and the second ranked gene was *Oprm1* (WLS = 81.89). Both are well-known targets of analgesic drugs, such as capsaicin patch and morphine. Other high-ranked genes presented in Table 4 also had well-known associations with pain disease because 19 of the 20 genes were included in the Pain Gene Database. A gene not included in this database was the FBJ osteosarcoma oncogene, ranked at 16.

**Table 4:** The top-20 ranked pain candidate genes gathered by our method

**Score**	**Gene**	**Description**
140.92	Trpv1	Transient receptor potential cation channel, subfamily V, member 1
81.89	Oprm1	Opioid receptor, mu 1
55.04	Trpa1	Transient receptor potential cation channel, subfamily A, member 1
39.56	Tacr1	Tachykinin receptor 1
38.78	Comt	Catechol-O-methyltransferase
31.04	P2rx3	Purinergic receptor P2X, ligand-gated ion channel, 3
30.96	Bdnf	Brain-derived neurotrophic factor
27.58	Scn9a	Sodium channel, voltage-gated, type IX, alpha
27.48	Ptgs2	Prostaglandin-endoperoxide synthase 2
26.33	Cnr1	Cannabinoid receptor 1 (brain)
23.67	Ngf	Nerve growth factor (beta polypeptide)
23.49	Tnf	Tumor necrosis factor
23.3	Asic3	Acid-sensing (proton-gated) ion channel 3
23.09	Scn10a	Sodium channel, voltage-gated, type X, alpha subunit
22.97	Tac1	Tachykinin, precursor 1
20.58	Fos	FBJ osteosarcoma oncogene
19.83	Gal	Galanin/GMAP prepropeptide
19.72	Calca	Calcitonin-related polypeptide alpha
19.41	Il6	Interleukin 6
19.06	Grin1	Glutamate receptor, ionotropic, N-methyl D-aspartate 1

### Comparison with other tools

**Figure 5:**
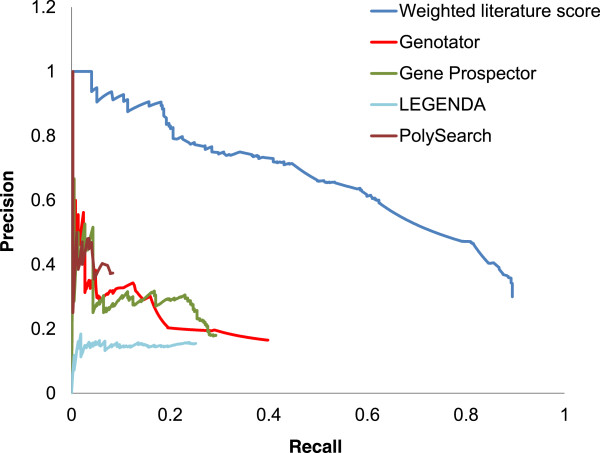
**Comparison of pain candidate gene prioritization performance with other publicly available tools.** Precision-recall plots show the performance of our method and of other publicly available tools. The precision and recall of other publicly available tools were calculated by the number of genes resulting from the use of the keyword “pain” using default parameters.

We compared our method against other publicly available tools using the pain gene set 2 and found the performance of our method to be superior, with a higher precision-recall curve across the entire range (Figure 5). Additionally, the maximum F-measure of our method was 0.61 which was higher than the other publicly available tools (Table 5); these had values of 0.23 (Genotator), 0.26 (Gene Prospector), 0.19 (LEGENDA), and 0.14 (PolySearch).

**Table 5:** Summary of the maximum F-measures for pain candidate genes from our method and those from publicly available tools

	**# of total gene**	**Max. F-measure**	**Rank at Max. F-measure**
Our method	1101	0.61	381
Genotator	892	0.23	544
Gene prospector	603	0.26	278
LEGENDA	601	0.19	601
PolySerach	83	0.14	83

One-hundred and sixty of 381 genes which were the number of genes at the rank of maximum F-measure were not extracted by other publicly available tools. Of these 160 genes, 94 were included in the pain gene set 2 (see Additional file 2). Acid-sensing ion channel 3 (ASIC3) which ranked at 13 (Table 4) is a typical example. ASIC3 is the most sensitive acid sensor in sensory neurons, and secondary mechanical hyperalgesia is not observed in knockout mice for this gene [25]. This channel is critical for the development of secondary hyperalgesia as measured by mechanical stimulation of the paw following muscle insult. Another example is the gene encoding L1 cell adhesion molecule, which was also identified solely by our method (ranked at 162, Additional file 1). This gene encodes a cell adhesion molecule that contributes to axonal outgrowth, guidance, and fasciculation in development, in addition to synapse formation and plasticity. This gene plays a role in the maintenance of thermal hyperalgesia following spinal cord injury in mice [26]. These results suggest that our method is more effective for the comprehensive gathering of pain candidate genes than publicly available tools.

### Case 2: Alzheimer’s disease

In order to evaluate the application of this method to other diseases, we conducted a gene search for AD. There is a clear and significant need for better therapy for AD. It is critical to understand the underlying molecular mechanism of AD for the development of novel treatments.

### Evaluation of scoring methods for gene prioritization

We evaluated the performance of scoring methods for gene prioritization for AD as we did in the case of pain. A total of 2178 genes were obtained from publications retrieved from a search using the MeSH term “Alzheimer Disease [Mesh:NoExp]” in PubMed. The prioritization scores of these genes were calculated using four methods. The recall of each method is shown in Figure 2B. The weighted literature score produced the best performance (p < .05). The higher ranked genes were predicted accurately with each method. The weighted literature score achieved better prediction accuracy with low ranking genes than that of other methods.

### Selection of AD-related MeSH terms

Figure 3 shows the recall of genes obtained from publications retrieved from searches for each set of MeSH terms using various similarity measures. The highest recall (0.886) was achieved using MeSH term set 6, which was constructed using MeSH term similarity calculated by the cosine similarity measure. This set comprised the combination of the MeSH terms “Alzheimer Disease”, “Amyloid beta-Peptides”, “Amyloid beta-Protein Precursor”, “Presenilin-1”, “Apolipoprotein E4”, “Plaque, Amyloid”, and “tau Proteins”. These seven MeSH terms were defined as AD-related MeSH terms.

### Gathering and prioritizing AD candidate genes

To assess the performance of our method, precision and recall for three gene sets were compared with AD gene set 2 at each gene rank. These sets were created using publications resulting from searches for AD-related MeSH terms, “Alzheimer’s disease” and “Alzheimer disease [MeSH]” in Pubmed.

Our method achieved a precision value of 0.04 and a recall value of 0.60 for 2810 genes. The top 20 ranked genes from our method are summarized in Table 6.

**Table 6:** The top-20 ranked AD candidate genes gathered by our method

**Score**	**Gene**	**Description**
1477.54	App	Amyloid beta (A4) precursor protein
1469.69	Apoe	Apolipoprotein E
794.48	Mapt	Microtubule-associated protein tau
560.37	Psen1	Presenilin 1
150.82	Bace1	Beta-site APP cleaving enzyme 1
105.30	Psen2	Presenilin 2
66.48	Snca	Synuclein, alpha (non A4 component of amyloid precursor)
62.84	Gsk3b	Glycogen synthase kinase 3 beta
58.73	Prnp	Prion protein
52.96	Bdnf	Brain-derived neurotrophic factor
46.54	Aplp2	Amyloid beta (A4) precursor-like protein 2
45.49	Serpine2	Serpin peptidase inhibitor, clade E (nexin, plasminogen activator inhibitor type 1), member 2
43.20	Mme	Membrane metallo-endopeptidase
39.21	Ide	Insulin degrading enzyme
38.82	Ncstn	Nicastrin
37.61	Cdk5	Cyclin-dependent kinase 5
37.08	Sorl1	Sortilin-related receptor, LDLR class A repeats-containing
36.96	Ace	Angiotensin I converting enzyme
35.06	Apbb1	Amyloid beta (A4) precursor protein-binding, family B, member 1 (Fe65)
32.46	Clu	Clusterin

The precision and recall values for 2940 genes from simple approach based on the ranking of the genes by the number of times the gene appears with keyword “Alzheimer’s Disease” were 0.04 and 0.63, respectively, whereas the precision and recall values for 2178 genes from simple approach with using the keyword “Alzheimer’s Disease [MeSH]” were 0.04 and 0.53, respectively. The maximum F-measures for AD-related MeSH terms, “Alzheimer’s Disease” and “Alzheimer Disease [MeSH]” were 0.24, 0.22, and 0.23 with gene ranks of 166, 134, and 194, respectively.

### Comparison with other tools

**Figure 6:**
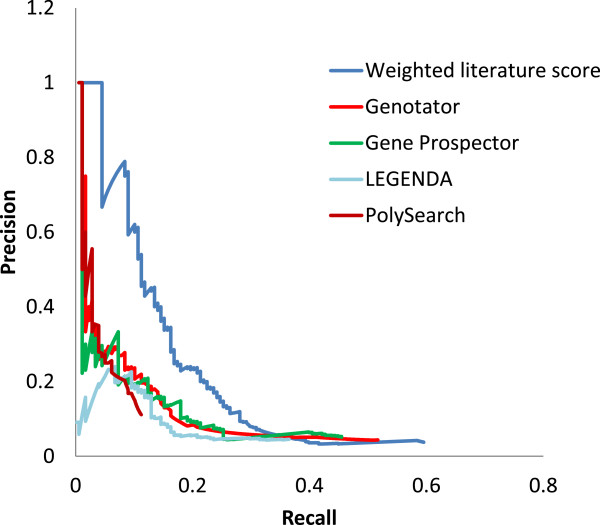
**Comparison of AD candidate gene prioritization performance with other publicly available tools.** Precision-recall plots show the performance of our method and of other publicly available tools. The precision and recall of other publicly available tools were calculated by the number of genes resulting from the use of the keyword “Alzheimer’s disease” using default parameters.

We also compared our method against other publicly available tools using AD gene set 2 and found the performance of our method to be superior (Figure 6). Additionally, the maximum F-measure of our method was 0.24, which was higher than other publicly available tools (Table 7). The publicly available tools had values of 0.15 (Genotator), 0.16 (Gene Prospector), 0.14 (LEGENDA), and 0.12 (PolySearch).

**Table 7:** Summary of the maximum F-measures for AD candidate genes from our method and those from publicly available tools

	**# of total gene**	**Max. F-measure**	**Rank at Max. F-measure**
Our method	2810	0.24	166
Genotator	2110	0.15	145
Gene prospector	1587	0.16	216
LEGENDA	1440	0.14	142
PolySearch	180	0.12	101

Two of 166 genes which were the number of genes at the rank of maximum F-measure were not extracted by other publicly available tools and were not included in AD gene set 2. These two genes were chemokine (C-X3-C motif) receptor 1 (CX3CR1) and Bcl2-associated X protein (BAX). Nonetheless, there are publications that provide evidence for an association between these genes and AD. CX3CR1, which ranked 132, is a key microglial pathway in protecting against AD-related cognitive deficits that are associated with aberrant microglial activation and elevated inflammatory cytokines [27]. BAX, which ranked 164, plays a role in neuronal cell death. Importantly, expression levels of these proteins are reportedly altered in vulnerable neurons in AD. The inhibition of Bax activity using either Bax-inhibiting peptide or *Bax* gene knockout significantly prevented oligomeric amyloid beta-induced neuronal cell death [28].

## Discussion

While several attempts have been made to develop methods for extracting and prioritizing disease candidate genes using bioinformatics techniques, a methodology for the inference of specific disease-relevant genes, such as pain or AD, has not yet been developed [10]. In this study, we developed computational methods to gather and prioritize the most likely candidate genes associated with specific disease. The features of our method include creating a set of disease related MeSH terms for the comprehensive retrieval of disease candidate gene related publications, and developing a novel prioritization score for improving ranking accuracy. The relationships between genes and publications were correctly obtained using the gene2pubmed database. There are other sources to find relationships between genes and publications such as Gene Reference Into Function (GeneRIF), which uses the gene-disease relation extraction system [29]. GeneRIF is a database in which human experts provide a brief summary of gene functions and make the connections between citations (PubMed) and NCBI Gene databases [30]. Although we used this index to obtain a relationship between genes and publications, we could not obtain a good performance (data not shown). Many methods for extracting knowledge from the literature using text mining are based on co-occurrence analysis of given keywords, which automatically extracts entity names from the text. However, automatic entity name recognition methods often incorrectly identify a significant portion of genes mentioned within the text [31,32] and consequently introduce noise and ambiguity into the extraction method. For example, in the case study of pain, the word TENS was recognized as a gene name by PolySearch and LEGENDA. However, in the field of pain research, TENS is usually used as an abbreviation for “transcutaneous electrical nerve stimulation”, a therapeutic strategy. To avoid this problem, our method used the gene2pubmed database which contains a list of associations between PubMed IDs and unambiguous gene identifiers.

By focusing on specific disease, we assembled disease-related MeSH terms to improve information retrieval from PubMed. In the case study of pain, a best set of MeSH terms, including “pain”, “pain measurement”, “nociceptor”, “pain threshold”, and “hyperalgesia”, was selected with the highest recall for pain gene set 1. Recall was reduced when MeSH terms (excluding pain-related MeSH terms) which appeared as frequently as “pain” (i.e., “posterior horn cells”, “ganglia, spinal”, “injections, spinal”, “physical stimulation”, and “formaldehyde”), were added to the set in the case of cosine similarity measure. This may be because these terms do not have a specific meaning for pain disease. “Posterior horn cells” and “ganglia, spinal” refer to tissue, while “injections, spinal” and “physical stimulation” refer to general methods, and “formaldehyde” identifies the general reagent. So, the genes that were not related to pain were extracted using these MeSH terms. Because pain-related MeSH terms belong to various MeSH categories, comprehensive gathering was achieved, which could not be accomplished by searching only the MeSH term “pain”. The example is the gene that encodes reticulon 4 (Rtn4), which was referenced in publications by the MeSH term “pain measurement”. This gene was assigned to GO term “GO: 0051930 regulation of sensory perception of pain”. The related publication indicated that Nogo-66, the 66-residue domain of Rtn4, may be related to a reduction in neuropathic pain following periphery nerve injury [33].

In the top-ranked 381 genes, 94 genes were not extracted by the other publicly available tools from the pain gene set 2; in addition, 121 pain candidate genes were not included in both pain gene set 1 and 2. An example of these 121 genes is FBJ murine osteosarcoma viral oncogene homolog (*FOS*) which ranked at 16, this has been extensively used as a marker for the activation of nociceptive neurons in the spinal cord [34]. The degree of spinal *c-Fos* expression was correlated with the extent of the pain-related behavior of carrageenan-injected rats [35]. Another example is sphingosine-1-phosphate receptor 1 gene (*S1PR1*) which ranked at 196. Sphingosine-1-phosphate (*S1P*) is a key regulator of the immune response. *S1P-* and inflammation-induced hypersensitivity is significantly reduced in mice with a conditional nociceptor-specific deletion in *S1PR1*[36]. Thus, S1P/S1PR1 signaling may be a key player in the onset of thermal hypersensitivity and hyperalgesia. These results suggest that our method was very effective for the comprehensive gathering of pain candidate genes.

To achieve high prioritization accuracy, we defined a weighted literature score. Several conventional methods have been used to calculate the co-occurrence frequency and appearance for the prioritization of each gene. Other text mining methods use the statistical p-value calculated from the number of publications [18]. However, the prioritization scores from these methods are likely to be overestimated, because several publications related to –omics analysis, such as microarray and genome sequence analysis, may cite many genes. We hypothesized that the contribution of these studies to each gene is relatively minor, and developed the weighted literature score to account for the number of genes studied in a given publication. The results of our study indicate that weighted literature scores improved performance compared with other scoring methods.

The results of our AD case study illustrate the applicability of our method to other diseases. However, the limitation is the lack of comprehensive applicability to other diseases. Disease-specific systems are able to exploit domain knowledge more thoroughly and thus achieve higher accuracy than general purpose systems, but the utility of these systems is not portable [37]. Our method is a semi-automated method for which a specialist’s knowledge is needed for GO and MP term selection. It is expected that adaptation to other diseases can also be promoted in the future if the retrieval of disease specific MeSH terms can be performed efficiently and automatically.

Another limitation of our method is that it will not discover novel disease candidate genes that are not cited in the disease-related literature. This includes studies that are newly registered in PubMed for which there would be no gene-PubMed ID relationship in GeneRIF or gene2pubmed and no MeSH term information. However, the accumulation of precise information from the literature by using our method may help to advance our understanding of disease mechanisms. It may also lead to the generation of novel hypotheses for understanding molecular mechanisms involved in disease when used in combination with further analyses such as network analysis [38]. We believe that our method for the comprehensive retrieval of disease candidate genes from the literature is a useful step toward understanding the mechanisms of disease.

## Conclusions

Our method, which involves the use of a set of disease-related MeSH terms and a weighted literature score, showed better performance than did other publicly available tools that extract general gene-disease associations.

The gene list obtained with our method would be beneficial for the study of disease mechanisms and would also provide a source of potential disease biomarkers and potential targets for novel therapies.

## Competing interests

The authors declare that they have no competing interests.

## Authors’ contributions

Both authors contributed to the design of the method and the analysis and interpretation of the data. TO implemented and carried out the study. Both authors read and approved the final manuscript.

## Supplementary Material

Additional file 1 A list of 1101 pain candidate genes prioritized by our method. Click here for file

Additional file 2 List of pain candidate genes by using our method, which were not extracted by publicly available tools. Click here for file
